#  Bioactivity-Guided Separation of an α-Amylase Inhibitor Flavonoid from *Salvia virgata*

**Published:** 2013

**Authors:** Bahman Nickavar, Leyla Abolhasani

**Affiliations:** Department of Pharmacognosy, School of Pharmacy, Shahid Beheshti University of Medical Sciences, Tehran, Iran.

**Keywords:** α-Amylase inhibitor, Diabetes mellitus, Chrysoeriol, *Salvia virgata*

## Abstract

It is now believed that the inhibition of carbohydrate hydrolyzing enzymes (CHEs) in the digestive tract can significantly prolong the overall carbohydrate digestion time and decrease the postprandial hyperglycemia after a meal. Therefore, inhibitors of CHEs can be useful therapeutic approaches in the management of diabetes mellitus, especially in the type 2, and complications associated with the disease. In our previous study, the ethanol extract of the aerial parts of Salvia virgata showed an inhibitory effect on pancreatic α-amylase in-vitro. Bioassay-guided fractionation of the extract using the α-amylase inhibitory assay led to the isolation and identification of an active flavone compound, chrysoeriol. The compound concentration dependently inhibited the α-amylase activity with an IC_50_ value of 1.27 (1.21-1.33) mM.

## Introduction

Diabetes mellitus is an endocrinal chronic disease characterized by elevated blood glucose levels (1-3). The control of hyperglycemia is critical in the management of diabetes mellitus since in long term, acute and chronic complications can occur (1, 4, 5). One goal of therapy for diabetic patients, especially non-insulin-dependent diabetes mellitus (type 2 diabetes), is the maintenance of normal blood glucose levels after a meal (postprandial hyperglycemia) (3, 6). A therapeutic approach for decreasing postprandial hyperglycemia is to retard and reduce the digestion and absorption of ingested carbohydrates by the inhibition of carbohydrate-hydrolyzing enzymes, such as *α*-amylase and/or *α*-glucosidases, in the digestive organs (1, 5, 7, 8). Therefore, there is a need to develop compounds with enzyme inhibitory activities, for which the medicinal plants may serve as potential sources (9, 10). 

The use of natural products as complementary approaches in existing medications for the treatment of diabetes mellitus is growing worldwide and many plants in different countries are known to have antidiabetic effects (11). Grover *et al. *reported that more than 1100 plant species have been used ethnopharmacologically or experimentally to treat diabetes mellitus (12). 

*Salvia *is one of the largest geniuses in Labiatae family. It comprises nearly 900 species throughout the world and 58 species in Iran (13, 14). Different species of *Salvia *have a long history of use for medical purposes in many countries (15, 16). On the other hand, investigations conducted on various species of *Salvia *show that the plants have wide and diverse biological activities especially antioxidant, anti-inflammatory, spasmolytic, antidiabetic, *etc *(13). For example, the hypoglycemic effect of *S. officinalis *that has long been used in Iranian traditional medicine for the treatment of diabetes, has been supported by some scientific studies (17-21). The scientific research has confirmed the potentially *α*-amylase inhibitory activities of few *Salvia *species. Nickavar *et al. *showed that the extracts from *S. virgata *and *S. verticillata *had inhibitory activities on pancreatic *α*-amylase (22). Furthermore, based on the study of Loizzo *et al*., *Salvia acetabulosa *was a potent *α*-amylase inhibitor (23).

However, no investigation has been ever done on the identification of *α*-amylase inhibitors from *Salvia *genus. The aim of the present work was to identify the antidiabetic compounds and *α*-amylase inhibitors from *Salvia virgata*.

## Experimental

*Plant material*

The aerial parts of *Salvia virgata *Jacq. (Synonym: *Salvia sibthorpii *Sibth. and Sm.) were collected from Tehran province during the flowering period in summer 2006. Voucher specimens were deposited at the Herbarium of the School of Pharmacy, Shahid Beheshti University of Medical Sciences, Tehran, Iran. The plant was dried at ambient temperature with active ventilation.

*Chemicals*

All of the chemical reagents used in this study were purchased from Sigma-Aldrich Chemical Co. (France) and/or Merck Company (Germany). Acarbose was obtained from Quimica Farmaceutica Bayer, S.A. (Barcelona).

*Extraction, chromatography and spectroscopy*

The dried and ground plant (250 g) was extracted with ethanol 90% three times by maceration method and then, the extract was concentrated *in vacuo*. The crude extract (12 g) was diluted with water and partitioned with *n*-C_6_H_12_, CHCl_3_ and EtOAc, successively. The inhibitory effects of the crude extract and all fractions were studied on *α*-amylase activity. The ethyl acetate fraction displayed the highest inhibitory activity. The fraction was subjected to more fractionation by column chromatography on silica gel using chloroform/ethyl acetate system as the eluent. The polarity of the eluent was increased by increasing the ratio of EtOAc during the process. Fraction F10 which showed a high inhibitory activity, was purified by repeated preparative layer chromatography on coated plates with silica gel (230-400 mesh) using CHCl_3_/EtOAc/HCOOH (45:45:10, v/v/v) as the best developing solvent system. Finally, fraction F10 yielded a pure active compound (53 mg).

The pure compound was identified on the basis of spectral and chromatographical studies. The UV-Vis spectrum was recorded on a Shimadzu UV-Vis spectrophotometer in methanol. The NMR spectra were taken on a Varian 400 spectrometer in DMSO-d_6_ and chemical shifts were recorded as *δ*-values. The EI-MS was obtained on a Finnigan-Mat spectrometer.

*α-Amylase inhibition test*

The *α*-amylase inhibitory activity was determined using the method described previously by Nickavar *et al*. (22). Briefly, 1 mL of the porcine pancreatic *α*-amylase enzyme solution (0.5 IU/mL) in 20 mM phosphate buffer (pH 6.9) was incubated with 1 mL of each test (at various concentrations) for 30 min. The reaction was initiated by adding 1 mL of 0.5% soluble potato starch solution and the mixture was incubated for 3 min at 25°C. Then, 1 mL of the color reagent (96 mM 3,5-dinitrosalicylic acid and 5.31 M sodium potassium tartrate in 2 M sodium hydroxide) was added and the mixture was placed in a water bath at 85°C. After 15 min, the reaction mixture was diluted with distilled water and the absorbance value was determined at 540 nm. Individual blanks were prepared for correcting the background absorbance. In this case, the color reagent solution was added prior to the addition of starch solution and the mixture was then placed in the water bath immediately. Controls were representative of the 100% enzyme activity. They were conducted in an identical fashion replacing tests with 1 mL of the solvent. Acarbose, a well-known *α*-amylase inhibitor, was used as positive control. The inhibition percentage of *α*-amylase was assessed by the formulae (1): 

$I_{á-\mathrm{amylase}}\left(\%\right)=100.(\frac{A_{\mathrm{control}}-A_{\mathrm{sample}}}{A_{\mathrm{control}}})$      (1)

Here, *A*control is the absorbance of each control and *A*sample is the net absorbance of each sample. The net absorbance of each sample was calculated by the equation (2): 

*A*_sample _=*A*_test_- *A*_blank_      (2) 

In this equation, *A*test is the absorbance of each test and *A*_blank _is the absorbance of each blank. 

The *I*_α-amylase_(%) for each sample was plotted against the logarithm of the sample concentration, and a logarithmic regression curve was established in order to calculate the IC_50_ valve. 

## Results and Discussion

The ethanol extract of *S. virgata *showed a dose-dependent inhibitory effect on the *α*-amylase activity [IC_50_ = 19.08 (18.61-19.56) mg/mL] (Table 1). 

**Table 1:** *α*-Amylase inhibitory activities and IC_50_ values of the aerial parts of *S. virgata *and its active compound chrysoeriol.

**Concentration**	**Inhibition (%)** ^a^	**IC**_50_^b^
**Extract (mg/mL)**		
**36.00**	83.69 ± 1.15	
**28.80**	74.28 ± 0.49	
**23.04**	70.92 ± 0.74	19.08 (18.61-19.56) mg/mL
**18.43**	30.83 ± 1.18	
**14.75**	18.37 ± 0.61	
**Chrysoeriol (mM)**		
**3.48**	96.28 ± 1.38	
**2.23**	70.62 ± 0.95	
**1.42**	58.98 ± 1.20	1.27 (1.21-1.33) mM
**0.91**	28.05 ± 0.80	
**0.58**	11.91 ± 0.85	

^a^ The data are expressed as mean ± SEM for five experiments in each group. ^b^The IC_50_ values were established by logarithmic regression curves with normalized data (using the computer software GraphPad Prism 3.02 for Windows) and presented as their respective 95% confidence limits

In order to identify the active components, solvent-solvent partition performed with *n*-C_6_H_12_, CHCl_3 _and EtOAc, successively. The ethyl acetate fraction revealed the highest activity so it was selected for further separation. The chromatographical analysis of the ethyl acetate fraction showed flavonoid compounds. The most active flavonoid compound was isolated as the pale yellow amorphous powder (53 mg). It had *ca *R _f_ = 0.7 on TLC (silica gel 60) with CHCl_3_/ EtOAc/HCOOH (45:45:10, v/v/v). The spectroscopic data for the compound were as follows: 

UV-Vis: λ_max_ (in CH_3_OH) = 266 (sh.), 354 nm

^1^H-NMR (400 MHz, in DMSO-d_6_), δ: 3.93 (3H, s, OCH_3_-3’), 6.18 (1H, br. s, H-6), 6.45 (1H, br. s, H-8), 6.58 (1H, s, H-3), 6.89 (1H, d, *J *= 8 Hz, H-5’), 7.37 (1H, br. s, H-2’), 7.40 (1H, d, *J *= 8 Hz, H-6’). 

^13^C-NMR (100 MHz, in DMSO-d_6_), δ: 54.8 (OCH_3_), 93.2 (C-8), 98.1 (C-6), 102.1 (C-3), 102.9 (C-10), 110.5 (C-2’), 115.7 (C-5’), 120.6 (C-6’), 130.4 (C-1’), 146.1 (C-3’), 148.8 (C- 4’), 154.4 (C-2), 155.7 (C-9), 161.3 (C-5), 167.6 (C-7), 171.0 (C-4). 

EI-MS (70 eV), *m*/*z *(*I *%): 300 (10%), 286 (82%), 153 (26%), 151 (20%). 

**Figure 1:**
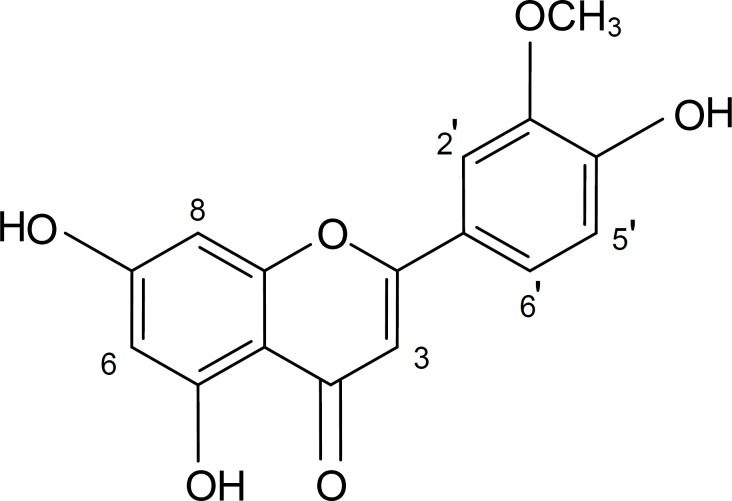
Chemical structure of chrysoeriol

The spectral data of the compound showed that it was chrysoeriol (Figure 1) and all of its data were matched with those reported in the literature (24, 25) .

**Figure 2:**
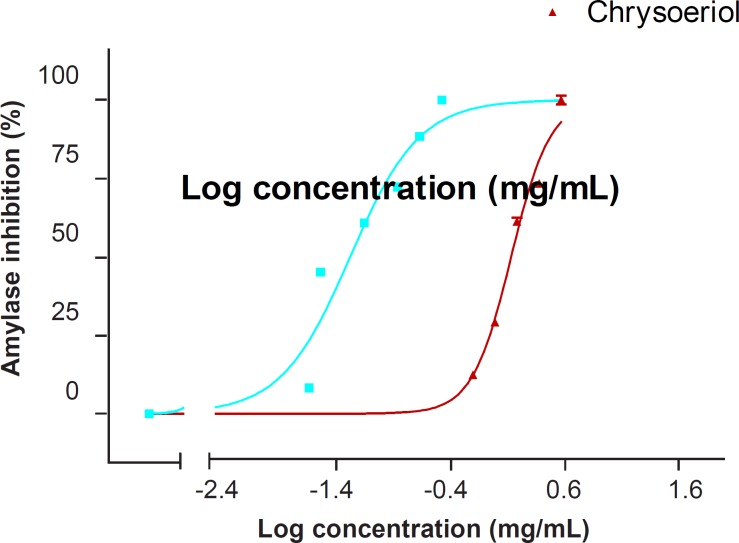
Dose-dependent inhibitory effect of chrysoeriol on the *α*-amylase activity. Each point represents the mean of five experiments and the vertical bars represent the SEM. The graphs were plotted using the computer software GraphPad Prism 3.02 for Windows

In this study, chrysoeriol inhibited *α*-amylase activity in a dose-dependent manner. The IC_50_ values for *α*-amylase inhibition by chrysoeriol and acarbose (as the positive control) were 1.27 (1.21-1.33) mM and 0.049 (0.042-0.056) mM, respectively (Figure 2 and Table 1).

The genus *Salvia *generally produces a variety of phenolic metabolites, especially flavonoids, which have received much attention due to their relevant biological properties (13). Phytochemical literature survey on *S. virgata *shows the occurrence of few hydroxycinnamic acid derivatives (such as rosmarinic acid, caffeic acid, *etc*.) and flavonoids (such as salvigenin, luteolin and its glycosides, luteolin 7,3’,4’-trimethyl ether, *etc*.) (13, 26, 27). On the other hand, chrysoeriol has already been isolated from few *Salvia *species including *S. candidissima*, *S. dorrii*, *S. lavandulaefolia*, *S. mirzayana*, and *S. palaestina *(13). However, to the best of our knowledge, this is the first report on the isolation and identification of chrysoeriol from *S. virgata *and the inhibitory effect of the compound on *α*-amylase activity.

## References

[1] Ali H, Houghton PJ, Soumyanath A (2006). α-Amylase inhibitory activity of some Malaysian plants used to treat diabetes; with particular reference to Phyllanthus amarus. J. Ethnopharmacol.

[2] Abesundara KJM, Matsui T, Matsumoto K (2004). α-Glucosidase inhibitory activity of some Sri Lanka plant extracts, one of which, Cassia auriculata, exerts a strong antihyperglycemic effect in rats comparable to the therapeutic drug acarbose. J. Agric. Food Chem.

[3] Li Y, Wen S, Kota BP, Peng G, Li GQ, Yamahara J, Roufogalis BD (2005). Punica granatum flower extract, a potent α-glucosidase inhibitor, improves postprandial hyperglycemia in Zucker diabetic fatty rats. J. Ethnopharmacol.

[4] Funke I, Melzig MF (2005). Phytotherapy in type 2 diabetes mellitus. Investigation of traditional herbal drugs as possible alpha-amylase inhibitors. Z. Phytother.

[5] Ye F, Shen Z, Xie M (2002). Alpha-glucosidase inhibition from a Chinese medical herb (Ramulus mori) in normal and diabetic rats and mice. Phytomedicine.

[6] Mai TT, Van Chuyen N (2007). Anti-hyperglycemic activity of an aqueous extract from flower buds of Cleistocalyx operculatus (Roxb.) Merr and Perry. Biosci. Biotechnol. Biochem.

[7] Dewi RT, Iskandar YM, Hanafi M, Kardono LBS, Angelina M, Dewijanti ID, Banjarnahor SDS (2007). Inhibitory effect of Koji Aspergillus terreus on α-glucosidase activity and postprandial hyperglycemia. Pak. J. Biol. Sci.

[8] Kwon YI, Jang HD, Shetty K (2006). Evaluation of Rhodiola crenulata and Rhodiola rosea for management of Type II diabetes and hypertension. Asia. Pac. J. Clin. Nutr.

[9] Etxeberria U, De La Garza AL, Campin J, Martnez JA, Milagro FI (2012). Antidiabetic effects of natural plant extracts via inhibition of carbohydrate hydrolysis enzymes with emphasis on pancreatic alpha amylase. Expert Opin. Ther. Tar.

[10] de Sales PM, de Souza PM, Simeoni LA, Magalhães PO, Silveira D (2012). α-amylase inhibitors: A review of raw material and isolated compounds from plant source. J. Pharm. Pharm. Sci.

[11] Hasani-Ranjbar S, Larijani B, Abdollah M (2008). A systematic review of iranian medicinal plants useful in diabetes mellitus. Arch. Med. Sci.

[12] Grover JK, Yadav S, Vats V (2002). Medicinal plants of India with anti-diabetic potential. J. Etyhnopharmacol.

[13] Lu Y, Yeap Foo L (2002). Polyphenolics of Salvia - A review. Phytochemistry.

[14] Mozaffarian V (1998). A Dictionary of Iranian Plants Names.

[15] Nickavar B, Asgarpanah J, Mojab F (2005). Volatile composition of the essential oil of Salvia hypoleuca Benth. Int. J. Aromather.

[16] Nickavar B, Kamalinejad M, Izadpanah H (2007). In-vitro free radical scavenging activity of five Salvia species. Pak. J. Pharm Sci.

[17] Amin G (2005). Popular Medicinal Plants of Iran.

[18] Eidi A, Eidi M (2009). Antidiabetic effects of sage (Salvia officinalis L.) leaves in normal and streptozotocin-induced diabetic rats. Diabetes Metab. Syndr.

[19] Eidi M, Eidi A, Zamanizadeh H (2005). Effect of Salvia officinalis L leaves on serum glucose and insulin in healthy and streptozotocin-induced diabetic rats. J. Ethnopharmacol.

[20] Hajzadeh MAR, Rajaei Z, Ghamami G, Tamiz A (2011). The effect of Salvia officinalis leaf extract on blood glucose in streptozotocin-diabetic rats. Pharmacologyonline.

[21] Lima CF, Azevedo MF, Araujo R, Fernandes-Ferreira M, Pereira-Wilson C (2006). Metformin-like effect of Salvia officinalis (common sage): Is it useful in diabetes prevention? Brit. J. Nutr.

[22] Nickavar B, Abolhasani L, Izadpanah H (2008). α-Amylase inhibitory activities of six Salvia species. Iran. J. Pharm. Res.

[23] Loizzo MR, Saab AM, Tundis R, Menichini F, Bonesi M, Piccolo V, Statti GA, de Cindio B, Houghton PJ (2008). In-vitro inhibitory activities of plants used in Lebanon traditional medicine against angiotensin converting enzyme (ACE) and digestive enzymes related to diabetes. J. Ethnopharmacol.

[24] Gu L, Wu T, Wang Z (2009). TLC bioautography guided isolation of antioxidant from fruit of Perilla frutescens var. acuta. LWT-Food Sci. Technolo.

[25] Awaad AS, Maitland DJ, Seliman GA (2006). Hepatoprotective activity of Schouwia thebica Webb. Bioorg. Med. Chem. Lett.

[26] Akkol EK, Goger F, Kosar M, Baser KHC (2008). Phenolic composition and biological activities of Salvia halophila and Salvia virgata from Turkey. Food Chem.

[27] Kosar M, Goger F, Baser KHC (2008). In-vitro antioxidant properties and phenolic composition of Salvia virgata Jacq. from Turkey. J. Agric. Food Chem.

